# Functional Characterization of a Spectrum of Genetic Variants in a Family with Succinic Semialdehyde Dehydrogenase Deficiency

**DOI:** 10.3390/ijms25105237

**Published:** 2024-05-11

**Authors:** Miroslava Didiasova, Samuele Cesaro, Simon Feldhoff, Ilaria Bettin, Nana Tiegel, Vera Füssgen, Mariarita Bertoldi, Ritva Tikkanen

**Affiliations:** 1 Institute of Biochemistry, Medical Faculty, University of Giessen, Friedrichstrasse 24, DE-35390 Giessen, Germany; miroslava.didiasova@biochemie.med.uni-giessen.de (M.D.); simon.feldhoff@bio.uni-giessen.de (S.F.);; 2 Department of Neuroscience, Biomedicine and Movement Sciences, University of Verona, Strada Le Grazie, 8, 37134 Verona, Italy; samuele.cesaro@univr.it (S.C.); ilaria.bettin@univr.it (I.B.); mita.bertoldi@univr.it (M.B.)

**Keywords:** neurotransmitter diseases, GABA, mitochondria, protein folding, chaperones, aldehyde dehydrogenases

## Abstract

Succinic semialdehyde dehydrogenase (SSADH) is a mitochondrial enzyme involved in the catabolism of the neurotransmitter γ-amino butyric acid. Pathogenic variants in the gene encoding this enzyme cause SSADH deficiency, a developmental disease that manifests as hypotonia, autism, and epilepsy. SSADH deficiency patients usually have family-specific gene variants. Here, we describe a family exhibiting four different SSADH variants: Val90Ala, Cys93Phe, and His180Tyr/Asn255Asp (a double variant). We provide a structural and functional characterization of these variants and show that Cys93Phe and Asn255Asp are pathogenic variants that affect the stability of the SSADH protein. Due to the impairment of the cofactor NAD^+^ binding, these variants show a highly reduced enzyme activity. However, Val90Ala and His180Tyr exhibit normal activity and expression. The His180Tyr/Asn255Asp variant exhibits a highly reduced activity as a recombinant species, is inactive, and shows a very low expression in eukaryotic cells. A treatment with substances that support protein folding by either increasing chaperone protein expression or by chemical means did not increase the expression of the pathogenic variants of the SSADH deficiency patient. However, stabilization of the folding of pathogenic SSADH variants by other substances may provide a treatment option for this disease.

## 1. Introduction

The catabolic pathway of the neurotransmitter γ-amino butyric acid (GABA) leads to the production of succinic semialdehyde (SSA), which is normally converted into succinate by a mitochondrial enzyme, succinic semialdehyde dehydrogenase (SSADH, reviewed in [1,2]). In the absence of SSADH activity, SSA and GABA accumulate in the cells and in the extracellular fluids [3,4]. In addition, a large fraction of SSA is converted into a toxic metabolite, γ-hydroxy butyric acid (GHB; reviewed in [1,2]). 

Impairment of SSADH activity is caused by mutations in the *ALDH5A1* gene, which resides on chromosome 6p22 and encodes the SSADH enzyme, the deficiency of which results in a rare genetic disease, SSADH deficiency (SSADH-D) [5,6]. The disease prevalence has been estimated to be about 1 in 460,000 [7]. The patients usually suffer from a varying degree of mental retardation, behavioral problems with autistic features, muscle hypotonia, and lack of speech. Some SSADH-D patients also have epileptic seizures that can be very severe and may result in sudden unexpected death from epilepsy [8,9,10,11,12]. Accumulation of the GABA metabolites is observed in the tissues and body fluids of the patients and can be used as a diagnostic tool. Recent years have brought about several interesting hypotheses on the molecular mechanisms of SSADH-D, with novel ideas for treatment options. The current understanding of SSADH-D and the molecular mechanism of this disease have recently been discussed in review articles that the readers should consider for more detailed information on this disease [2,12,13,14]. 

SSADH-D is a recessively inherited disease in which a large spectrum of pathogenic *ALDH5A1* gene variants have been described, and the sequencing of the *ALDH5A1* gene is usually performed to verify the diagnosis [1,15,16,17]. However, there is only a poor genotype–phenotype correlation even within a single family. No major mutations that would be present in a high fraction of patients are known, and most patients have their private or family-specific pathogenic gene variants. In addition to a growing list of various pathogenic mutations in the *ALDH5A1* gene, a number of presumably benign single nucleotide polymorphisms (SNPs) are listed in genetic databases, such as ClinVar and GnomAD [18,19]. This poses a problem for the genetic diagnosis of SSADH-D, as it is not always clear how significant a certain gene variant is for the impairment of SSADH activity. 

One such *ALDH5A1* variant is c.538T>C, p.His180Tyr (SNP rs2760118), which is very common in the general population, with an allelic frequency of 0.348 [20]. The His180Tyr variant was described to be benign, with only mildly reduced or near-normal enzyme activity in overexpression systems [15,16,21]. However, it can exacerbate the effect of other amino acid substitutions present in the same allele of the *ALDH5A1* gene, such as the Pro182Leu (c.545C>T) variant. Pro182Leu alone is also not very harmful, but together with His180Tyr, it shows a more severe effect on the SSADH enzyme activity [15]. Therefore, it is important to properly characterize all variants, including the seemingly benign ones, that are present in a specific SSADH-D patient, before conclusions about the pathogenicity of the variants can be made. 

In the present study, we describe an SSADH-D family with four *ALDH5A1* gene variants, including the known His180Tyr and Cys93Phe variants [15] and two previously uncharacterized variants. We provide a thorough molecular and structural analysis of these variants, showing that Val90Ala and His180Tyr are benign variants, whereas Cys93Phe and Asn255Ala are pathogenic and result in a profound loss of SSADH protein expression and impairment of enzyme activity. We also show that the treatment of cells expressing these variants with substances that enhance protein folding does not result in an improvement of SSADH expression or activity in the case of the above-mentioned pathogenic variants. Our data show that it is important to understand the molecular consequences of the potentially pathogenic variants as well as their combinations so that personalized precision therapies targeting patient-specific variants can be developed [13]. 

## 2. Results

### 2.1. SSADH Deficiency Patient Description

A male infant was born in-term as the first child of a non-consanguineous German couple without any family history of SSADH-D. According to the family, a mild developmental delay and muscle hypotonia were observed in the patient from early childhood on. At the age of about 18 months, urine analysis revealed an increased amount of GHB, a hallmark of SSADH-D, and the diagnosis of SSADH-D was confirmed by genetic sequencing by a diagnostic company. 

**Figure 1:**
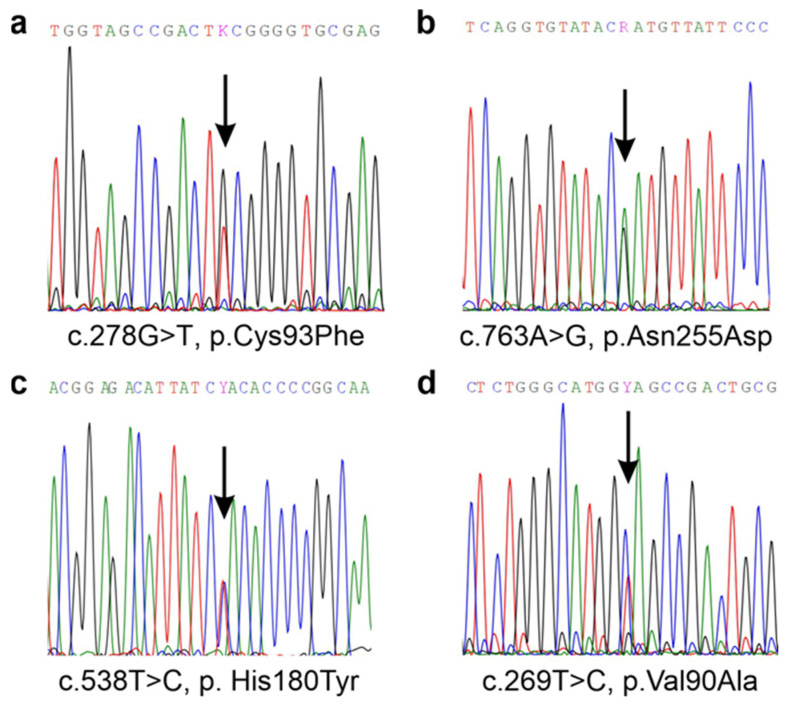
Sequencing results of the SSADH-D patient family revealed four different genetic variants that were all present in a compound heterozygous form. (**a**) Paternal variant c.278C>T, p.Cys93Phe and (**b**) maternal variant, c.763A>G, p.Asn255Asp that were found in the patient. (**c**) In the maternally inherited *ALDH5A1* allele, an additional variant, c.538T>C, p.His180Tyr was detected. (**d**) A variant of unknown significance, c.269T>C, p.Val90Ala, was detected in the mother but not in the patient.

Upon the original genetic diagnosis in 2014, the patient was found to be heterozygous for two missense variants in the *ALDH5A1* gene. The paternal variant, c.278C>T, p.Cys93Phe (Figure 1a), is a known pathogenic SSADH variant [15], whereas the maternal variant, c.763A>G (p.Asn255Asp, Figure 1b) was a novel one, i.e., a variant of unknown significance (VUS). However, a pathogenic variant in the same site, p.Asn255Ser, has already been described in an SSADH-D patient [15]. Thus, it was likely that the Asn255Asp variant would also be a pathogenic one, although its impact in terms of changes in polarity and steric features may be less than that of Asn255Ser. In addition, two SNPs, rs2760118 (c.538T>C, p.His180Tyr; Figure 1c) and rs1272389025 (c.269T>C, p.Val90Ala; Figure 1d) were present in the maternal sequencing results [20]. Both SNPs were deemed to be without clinical relevance by the original diagnostic company, even though the His180Tyr variant has been shown to exacerbate the effect of SSADH missense variants that are present in the same allele [15], and Val90Ala has not been studied before. 

Dermal fibroblast cultures were established from a punch biopsy of the patient for functional studies of the genetic *ALDH5A1* variants. Upon further sequence analysis, the His180Tyr variant indeed turned out to be present in the same maternal allele as the Asn255Asp variant (thus designated as His180Tyr/Asn255Asp), whereas the Val90Ala variant resided in the other maternal allele that was not inherited by the patient, but was later found in the younger sister. 

### 2.2. Molecular Characterization of the SSADH Deficiency Patient Variants

**Figure 2:**
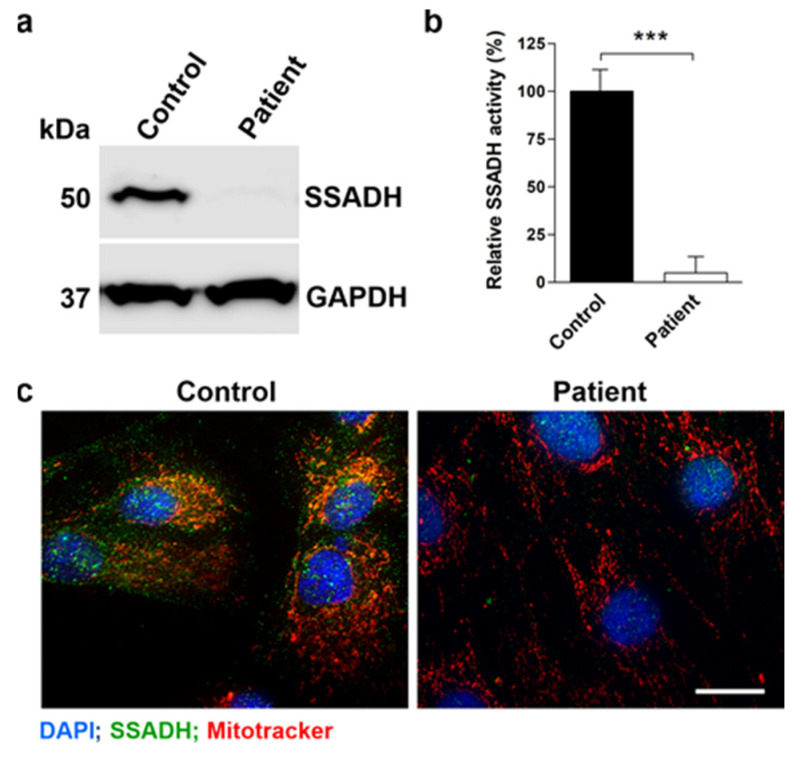
SSADH protein expression, activity, and localization in control and patient fibroblast cultures. (**a**) SSADH protein expression in control and patient fibroblasts. GAPDH was used as a loading control. Representative Western blots from three independent experiments are shown. (**b**) Relative SSADH enzyme activity was assessed by a fluorometric assay. Control fibroblast activity was set to 100%. Data are expressed as mean values ± SD (*n* = 3). *** *p* ≤ 0.001 (Student’s *t*-test). (**c**) Fluorescent images of control and patient fibroblasts stained with an anti-SSADH antibody (green) and MitoTracker (red) for visualization of mitochondria. Scale bar 20 μm.

Western Blot analysis with anti-SSADH antibody revealed barely any detectable SSADH expression in the patient fibroblasts as compared to control fibroblasts (Figure 2a). Furthermore, SSADH activity was significantly reduced in the patient fibroblasts (Figure 2b), suggesting that the missense variants are indeed pathogenic and cause a destabilization of the SSADH protein, resulting in no or low expression of the SSADH protein. Consistently, immunostaining of the patient fibroblasts for the SSADH protein revealed very little SSADH signal as compared to the control fibroblasts (Figure 2c, green signals). 

**Figure 3:**
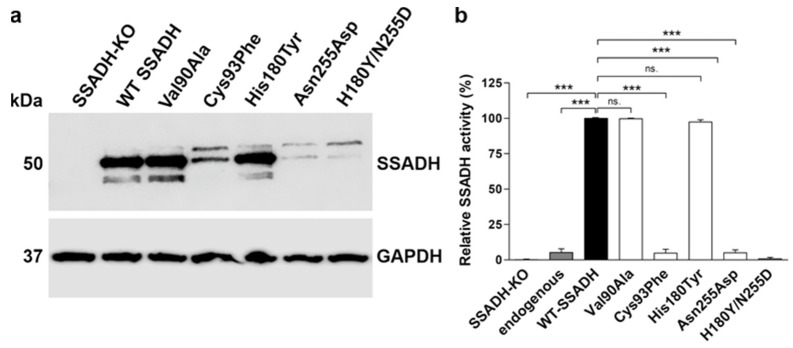
In vitro characterization of SSADH deficiency patient variants in a transient overexpression system. HEK-293T *ALDH5A1* knockout (SSADH-KO) cells were transiently transfected with pcDNA 3 vector carrying either WT or Val90Ala, Cys93Phe, His180Tyr, Asn255Asp or His180Tyr/Asn255Asp SSADH variant. After 48 h, cell lysates were prepared and (**a**) subjected to Western blotting. GAPDH was used as a loading control. Representative Western blots are shown (*n* = 3). (**b**) SSADH activity of SSADH-KO HEK-293T cells overexpressing WT SSADH was set to 100%; the variant activities are expressed as % WT. Data are expressed as mean values ± SD (*n* = 3). *** *p* ≤ 0.001, ns. = non-significant (one-way ANOVA followed by Tukey’s post-hoc test).

As the pathogenic nature of the Val90Ala variant and the Asn255Asp variant and its combination with His180Tyr was unclear, biochemical characterization of all individual genetic *ALDH5A1* variants found in the family was performed at the protein level. For this, the variants were cloned in the pcDNA3 vector and transiently overexpressed in HEK293T *ALDH5A1* knockout cells [22] (Figure 3). The mature, processed SSADH is detected at about 50 kDa, whereas the unprocessed precursor protein that contains the mitochondrial targeting signal is located at 52 kDa. In addition, signals below 50 kDa are visible for the variants showing high expression, and they are likely to represent degradation products. The variants Val90Ala and His180Tyr exhibited a protein amount comparable with the wildtype (WT) SSADH protein, whereas Cys93Phe, Asn255Asp, and His180Tyr/Asn255Asp were expressed in very low amounts (Figure 3a). Consistently, the SSADH activity of the latter three variants was barely measurable (below 5% of the WT), whereas the activities of Val90Ala and His180Tyr were comparable to the WT SSADH activity (Figure 3b). Therefore, Cys93Phe, Asn255Asp, and His180Tyr/Asn255Asp are obviously pathogenic variants that result in a profound loss of both SSADH protein level and activity.

**Figure 4:**
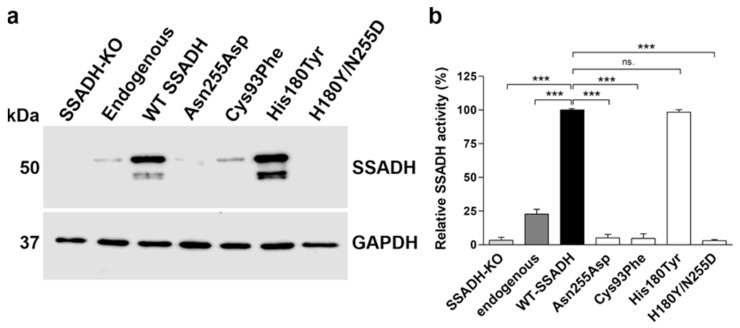
In vitro characterization of the SSADH-D patient variants in a knock-in system. (**a**) SSADH protein expression and (**b**) relative SSADH enzyme activity in SSADH-deficient Flp-In^TM^-293 cells (SSADH-KO), Flp-In^TM^-293 (with endogenous SSADH), and SSADH-KO Flp-In^TM^-293 cells with genomic integration of either the WT SSADH or one of the SSADH variants (Asn255Asp, Cys93Phe, His180Tyr or Asn255Asp/His180Tyr). (**a**) Expression of SSADH variants. GAPDH was used as a loading control. Representative Western blots from three independent experiments are shown. (**b**) SSADH enzyme activity of SSADH-KO Flp-In^TM^-293 cells stably expressing WT SSADH was set to 100%, and the variant activities are expressed as % WT. Data are expressed as mean values ± SD (*n* = 3). *** *p* ≤ 0.001, ns. = non-significant (one-way ANOVA followed by Tukey’s post-hoc test).

Since high-degree, transient overexpression may not always fully recapitulate the effects of natural missense variants of SSADH [16], HEK293 cell lines with a genomic knock-in were produced for the WT, His180Tyr and the three pathogenic variants. First, the endogenous *ALDH5A1* gene was knocked out in Flip-In^TM^-293 cells. Thereafter, the SSADH WT and variant coding regions cloned in pcDNA5-FRT plasmid were inserted in the genomic Flp Recombination Target (FRT) site using the FRT recombinase activity of the Flip-In system [23]. The expression of the stable knock-in variants (Figure 4) was highly consistent with the transient expression data (Figure 3), with the His180Tyr variant showing a WT-like expression, whereas the potentially pathogenic variants Cys93Phe, Asn255Asp, and His180Tyr/Asn255Asp exhibited only very low amounts of the SSADH polypeptide (Figure 4a). Measurement of the relative SSADH activity revealed a WT level of activity for the His180Tyr variant (Figure 4b), supporting the previous findings showing that this variant is, per se, not pathogenic [15,16,21]. In contrast, Cys93Phe, Asn255Asp, and His180Tyr/Asn255Asp showed only background activity comparable with the SSADH knockout cells (Figure 4b).

### 2.3. Bioinformatic Analyses of the SSADH Variants

**Figure 5:**
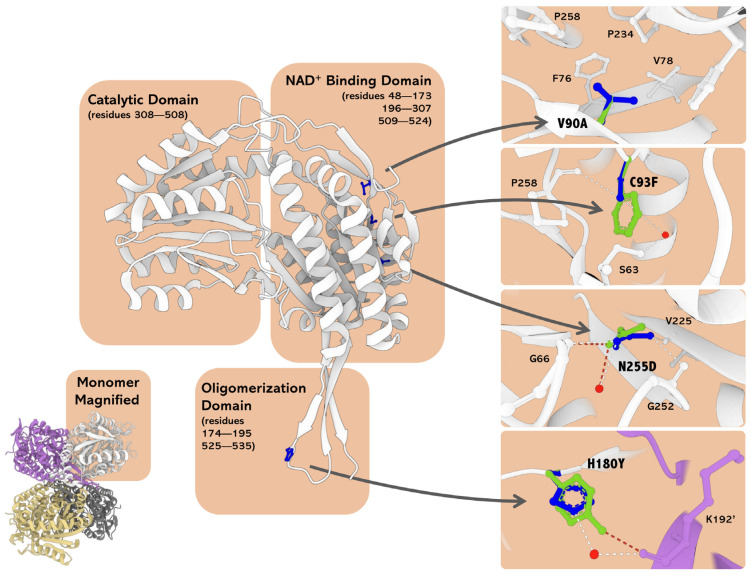
Oligomeric and monomeric structures of human SSADH (PDB 2W8N) and in silico mutagenesis. The monomer is represented as ribbons, and the mutated residues are shown as blue balls and sticks. In silico mutagenesis of the selected residues was carried out with the software PyMOL (The PyMOL Molecular Graphics System, Version 2.2.3; Schrödinger, LLC, New York, NY, USA). The four insets represent the microenvironment and interaction network changes induced by the substituted amino acids (i.e., V90A, C93F, N255D, and H180Y, from top to bottom) with respect to the WT. The variant residues (in green) are superimposed on the corresponding WT residue (in blue). The dashed lines represent the new and lost contacts formed by these residues in red (variant) and white (WT). Red dots represent water molecules. Since the residue His180 belongs to the oligomerization domain, the neighboring subunit (light purple) is shown for H180 and the H180Y variant. The image was built by MOL* [26].

For structural in silico assessment of the SSADH variants, a bioinformatics analysis was performed. The amino acids altered in the SSADH variants reside in two different domains of the SSADH protein: in the NAD^+^ binding or the oligomerization domains (Figure 5). More precisely, Val90 and Cys93 are part of an α-helix at the surface of the NAD^+^ binding domain. Cys93 is engaged in multiple hydrogen bonds with the surrounding residues, stabilizing the monomer structure. In particular, hydrogen bonds with the backbone carbonyl of Pro258 and the hydroxyl moiety of Ser63 are formed, the latter of which is mediated by a water molecule (Figure 5). The substitution of Cys93 with Phe causes a loss of such interactions, thus resulting in profound structural impairments within the domain, as also shown before [17]. The Val90Ala substitution, given the conservation of the apolar chemical nature, does not change the hydrophobic microenvironment, in which Val90 is placed in a hydrophobic contact with Phe76, Val78, Pro234, and Pro258 (Figure 5). Thus, this variant, found in combination with the pathogenic His180Tyr/Asn255Asp in the mother of the patient, who is a healthy carrier, is not likely to be pathogenic. 

The highly conserved Asn255 (Consurf value = 8) maps in a central stacked β-sheet in the proximity of the NAD^+^ binding groove. It interacts with the residues Gly252 and Val225 that belong to the same domain but are deeply buried within the NAD^+^ binding cleft. The Asn255Asp substitution breaks these interactions, and new H-bonds with the backbone amino moiety of Gly66 are formed (Figure 5). These modifications may produce a structural domino effect by displacing the residues 252–282 that build the NAD^+^-binding cleft. Even a loss of the tetrameric SSADH structure may be induced since the stacked β-sheet containing Asn255 is a fundamental structural element for the assembly of the SSADH oligomer [24]. 

His180 is a residue with an average degree of conservation (Consurf value = 4), and it resides in the oligomerization domain essential for the formation of the functionally active SSADH tetramer [24]. Through a water molecule, its imidazole moiety contacts the side chain of Lys192’ in the neighboring monomer (the prime denotes residues belonging to the neighboring subunit within the tetramer), thus contributing to the oligomer architecture (Figure 5). Upon substitution of His180 with Tyr, this interaction is preserved, as it is mediated by the hydroxyl group of the Tyr side chain at 2.6 Å distance from the ε-amino group of Lys192’ (Figure 5). Thus, the structural effects of the His180Tyr substitution are modest. Since His180 resides at the interface between the adjacent monomers, the predicted stabilization/destabilization energy of the His180Tyr variant, as compared to the WT protein, was predicted by the computational tool BindProfX [25]. The His180Tyr substitution only slightly alters the interface, with a measured ∆∆G value of +0.63 kcal/mol.

### 2.4. Spectroscopic and Kinetic Features of the SSADH Variants

According to our data, Val90Ala is a non-pathogenic variant, and Cys93Phe has already been characterized elsewhere [15,17,24]. Therefore, these two variants were not characterized further. However, the structural and functional features of His180Tyr and His180Tyr/Asn255Asp variants were analyzed using recombinant, purified SSADH protein species. Further analysis of the recombinant Asn255Asp variant was prevented by the fact that it turned out to be an insoluble species, at least in the *E. coli* bacterial expression system.

**Figure 6:**
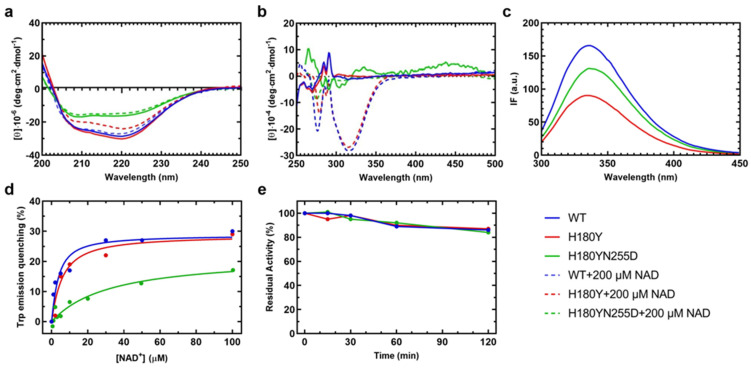
Spectroscopic signals and apparent equilibrium dissociation constants for NAD^+^, and time course of reactions of WT (blue line), H180Y (red), and H180Y/N255D (green) SSADH variants. (**a**) Far-UV and (**b**) near-UV/visible circular dichroism (CD) spectra of the WT SSADH and the variants. Enzyme concentration was 0.1 mg/mL for far-UV and 1 mg/mL for near-UV/visible CD in 100 mM potassium phosphate buffer, pH 8. Spectra of unliganded (straight line) and NAD^+^-bound (dashed line) enzyme species are reported (**c**) Emission fluorescence intensity of WT and the variant SSADH species at 0.1 mg/mL, following excitation at 280 nm in 100 mM potassium phosphate buffer, pH 8. (**d**) Data for the calculation of the apparent equilibrium dissociation constant (K_D_) for NAD^+^, calculated as reported in chapter 4.15. (**e**) Loss of residual activity over time of the dehydrogenase reaction catalyzed by the WT and the variant SSADH species. The activity of each species at the beginning (timepoint 0) was set to 100%, and the decline was monitored over time.

The far-UV circular dichroism (CD) spectrum (Figure 6a) of the His180Tyr variant (0.5 µM tetramer) is similar to that of the WT (0.5 µM tetramer). It presents characteristic minima at 208 and 222 nm, representative of a predominantly α-helical conformation, slightly altered in the presence of the coenzyme NAD^+^ for both the WT and the His180Tyr species (Supplementary Table S1). Interestingly, NAD^+^ binding to the WT species decreases the percentage of α -helices and increases that of β-sheets. The relative proportion of secondary structure elements evaluated for the His180Tyr variant in the presence of NAD^+^ shows an increase in other secondary structure elements at the expense of β-sheets. It is a common feature that coenzyme binding causes modifications in the secondary structure spectra for NAD^+^-dependent dehydrogenases [27], which can be interpreted as a sign of NAD^+^ binding. The double variant His180Tyr/Asn255Asp exhibits a different far-UV CD spectrum that is less intense than those of the WT and His180Tyr, with an altered ratio of 208 nm/222 nm minima. No modifications were observed in the presence of NAD^+^, raising the question of how effective the coenzyme binding to this enzymatic species is (Figure 6a).

The binding of NAD^+^ stabilizes WT and His180Tyr SSADH, as revealed by the increase of about 4 °C in the melting temperature (T_M_) values evaluated by monitoring the change in the dichroic signal at 222 nm in both species (Supplementary Table S1). The T_M_ of the His180Tyr/Asn255Asp variant is 51.5 ± 0.2°C, a value similar to that of the other SSADH species in the absence of the coenzyme and unaffected by the presence of 200 µM NAD^+^. 

The tetrameric molecular structure of WT and His180Tyr SSADH was assessed by size exclusion chromatography, with concentrations of 0.1 to 5 mg/mL (Supplementary Figure S1), confirming that the amino acid change in the oligomerization domain does not affect the overall quaternary structure of the protein. 

The near-UV and visible CD spectrum of the WT SSADH presents dichroic signals related to some aromatic amino acid(s) that reside in an asymmetric environment (Figure 6b). In the presence of NAD^+^, a negative peak at 330 nm appears, reflecting the binding of the cofactor, with a slight alteration of the region around the aromatic amino acids. The His180Tyr variant shows a highly similar spectral behavior as the WT without NAD^+^. The CD signals of the His180Tyr/Asn255Asp variant present slight alterations as compared to the WT, both in the absence or presence of NAD^+^, suggesting that the coenzyme binding pocket is affected and the complex is not dichroic in the presence of NAD^+^, possibly due to an alteration in the microenvironment (Figure 6b).

The intrinsic fluorescence emission spectra reveal differences in the intensity values with a similar λ_max_ = 332 nm for the WT and the His180Tyr variant and a red-shift (λ_max_ = 335 nm) for the His180Tyr/Asn255Asp variant (Figure 6c), indicating some alteration in the hydrophobic protein core, which is more pronounced in the case of the His180Tyr/Asn255Asp variant. The affinity for NAD^+^ was evaluated by measuring the change in the intrinsic fluorescence emission following excitation at 280 nm at increasing coenzyme concentrations (Figure 6d). The apparent equilibrium dissociation constants, K_D_, were calculated as described in chapter 4.15. WT and His180Tyr variants present similar K_D_ values (2.8 ± 0.3 µM for WT and 3.0 ± 1 µM for His180Tyr; Figure 6d), while the His180Tyr/Asn255Asp variant exhibits a strongly decreased affinity for the coenzyme, with a measured K_D_ of 33 ± 13 µM, a value 10-fold higher than the WT (Figure 6d).

When assayed for the residual enzyme activity in the presence of 10 µM SSA and 500 µM NAD^+^, the His180Tyr and His180Tyr/Asn255Asp variants show different degrees of activity, with the His180Tyr variant retaining 83% (54 s^−1^) of the WT activity (65 s^−1^). In contrast, the activity of the His180Tyr/Asn255Asp variant dramatically drops to 0.1% (0.09 s^−1^), suggesting that a profound structural change is induced that leads to catalytic impairment. Interestingly, when the stability of the enzymatic species (WT and variants) is followed by evaluating the relative activity loss over time (Figure 6e), all species behave in a similar manner for at least two hours, corroborating that the Asn255Asp substitution induces a structural impairment deeply affecting the catalysis.

### 2.5. Folding Therapy Approaches for the SSADH Variants

The folding of pathogenic missense variants can, in some cases, be improved by chemical or pharmacological chaperones [28,29,30,31]. A further possibility to enhance chaperone-mediated folding is small molecules, such as celastrol and arimoclomol, which increase the expression of the members of the Heat shock protein 70 (Hsp70) family [28,29,30,31]. Not only chaperone proteins but also pharmacological chaperones (PCs, acting by directly binding and stabilizing their specific target proteins) and chemical chaperones that show a generally positive effect on protein folding can improve the folding of pathogenic variants [29,30]. Betaine (tri-methyl glycine) is a small molecule that has been shown by us and others to function as a PC substance for pathogenic variants of proteins residing in peroxisomes or lysosomes [32,33]. However, it is not known if betaine could directly bind to SSADH. Betaine is also used as a chemical chaperone to support protein folding and stability during protein purification and storage. In addition, betaine has several beneficial effects that can improve the state of cells, such as osmotic and lipotrophic effects [34]. 

**Figure 7:**
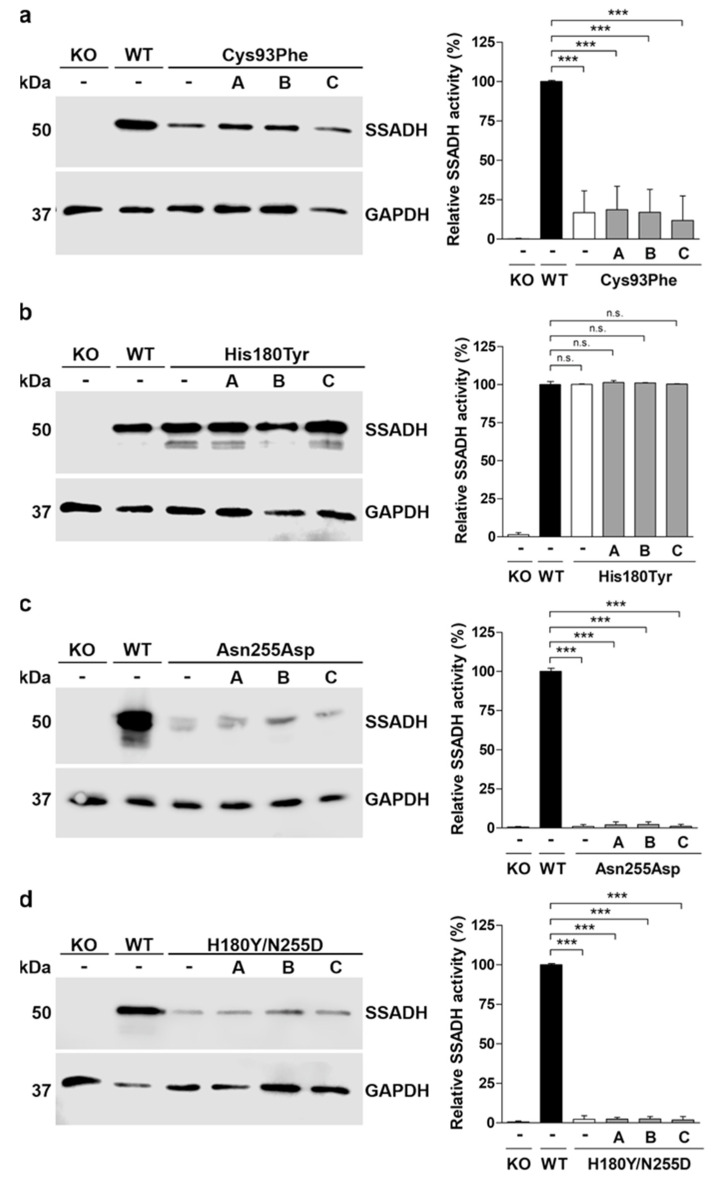
Treatment of knock-in cells stably expressing the SSADH patient variants. (**a**–**d**) SSADH-deficient Flp-In^TM^-293 cells (KO) stably expressing one of the SSADH variants (Cys93Phe, His180Tyr, Asn255Asp or Asn255Asp/His180Tyr) were treated with 50 µM arimoclomol (A), 10 mM betaine (B), or 250 nM celastrol (C). After 24 h, cell lysates were prepared and subjected to Western blotting (left panels) and SSADH enzyme activity assay (right panels). GAPDH was used as a loading control for Western blots. Representative Western blots are shown (*n* = 3). SSADH activity of SSADH-KO Flp-In^TM^-293 cells stably expressing WT SSADH was set to 100%, and the variant activities are expressed as % WT. Data are expressed as mean values ± SD (*n* = 3). *** *p* ≤ 0.001, n.s. = non-significant (one-way ANOVA followed by Tukey’s post-hoc test).

Due to these potentially beneficial effects, stable knock-in cells expressing the SSADH variants were treated with arimoclomol, betaine, or celastrol for 24 h. The effect of these substances on the SSADH variants was assessed by Western blot and SSADH activity measurement (Figure 7). Unfortunately, none of the substances showed a significant effect on SSADH protein level or activity (Figure 7a–d). 

**Figure 8:**
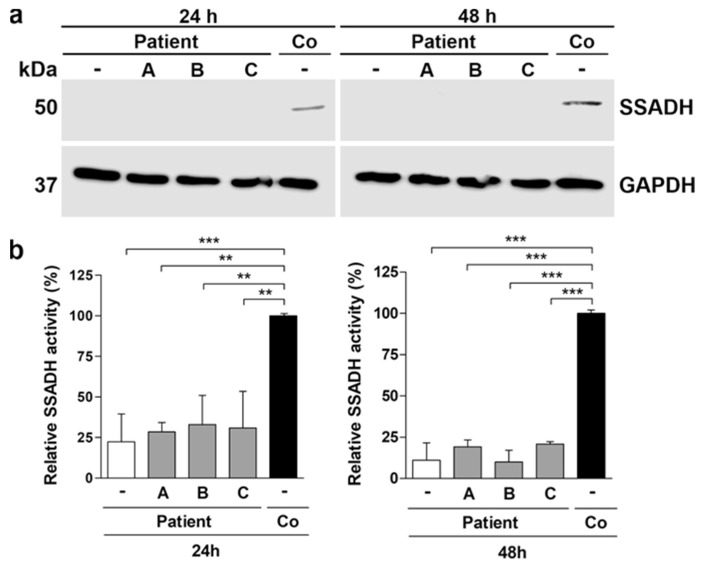
Treatment of SSADH deficiency patient fibroblasts. Patient fibroblasts were treated either with 50 µM arimoclomol (A), 10 mM betaine (B), or 250 nM celastrol (C) and were compared to untreated control fibroblasts (Co). After 24 h or 48 h, cell lysates were prepared and subjected to (**a**) Western blotting and (**b**) SSADH enzyme activity assay. GAPDH was used as a loading control in the Western blots. Representative Western blots are shown (*n* = 3). (**b**) SSADH activity of untreated control fibroblasts was set to 100%, and the relative SSADH activities in the patient cells were expressed as % WT. Data are expressed as mean values ± SD (*n* = 3). ** *p* ≤ 0.01; *** *p* ≤ 0.001 (one-way ANOVA followed by Tukey’s post-hoc test).

Similar data were obtained with the fibroblasts of the SSADH deficiency patient treated for 24 h or 48 h with the above substances (Figure 8). In the untreated patient fibroblasts, expression of SSADH was barely detectable. Neither arimoclomol, betaine, nor celastrol were able to significantly increase the expression or activity of SSADH in the fibroblasts of the patient (Figure 8a,b). After 48 h of treatment, arimoclomol and celastrol produced a minor increase in SSADH activity, but the difference to untreated cells failed to become significant (Figure 8b, right). Therefore, the three compounds tested do not seem to show any clear beneficial effect on the expression and activity of the pathogenic SSADH variants. 

## 3. Discussion

**Figure 9:**
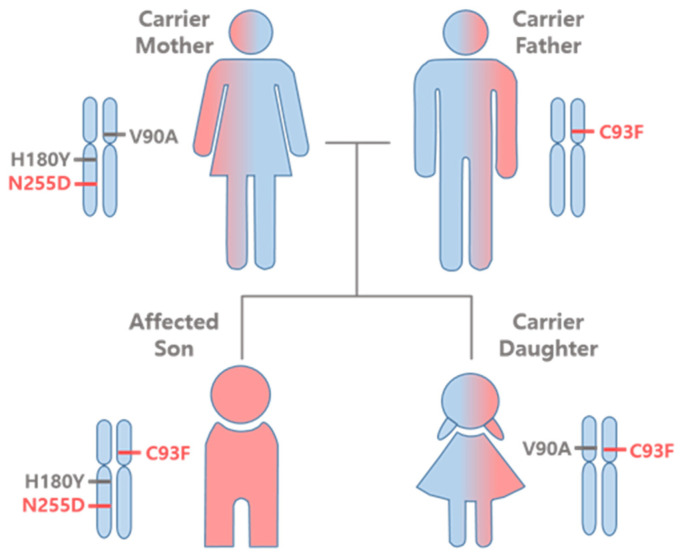
Variants found in the members of the SSADH-D patient family. Pathogenic *ALDH5A1* variants on their respective chromosomes are shown in red, benign variants in grey.

In the present study, we have provided a thorough structural and functional characterization of the SSADH variants found in the family of a patient with SSADH-D. A schematic summarizing the alleles found in the family is shown in Figure 9. Upon the original genetic diagnosis, two of these variants, Val90Ala and His180Tyr, were declared as SNPs without any clinical relevance, even though Val90Ala was a novel, uncharacterized variant (i.e., a VUS), and His180Tyr was known to exacerbate the effect of further amino acid substitutions [15,16,21]. The younger sister of the index patient in this family was later found to be a heterozygous carrier of Cys93Phe (paternal) and Val90Ala (maternal) variants. Therefore, it was important to characterize the molecular effects of the variants found in the family. The Val90Ala variant showed a WT-like expression and activity, thus confirming its non-pathogenic nature. 

The Cys93Phe and Asn255Asp variants, located within the NAD^+^ binding domain, exhibited not only a highly reduced SSADH enzyme activity but also a very low level of protein expression in eukaryotic cells, including patient fibroblasts. Akaboshi et al. have experimentally shown that the Cys93Phe variant shows a very low (3% of WT) activity in a transient overexpression system, but the protein expression level was not assessed [15]. Pop et al. have studied another substitution at this site, Cys93Arg, showing that this variant exhibited only a very low residual enzyme activity, but they did not study the protein expression level [16]. Exchange of Cys93 to Phe (or Arg) perturbs several hydrogen bonds within the cofactor binding domain, resulting in structural destabilization of these SSADH variants, as also previously predicted [17,24]. In addition, the Cys93Phe exchange also causes a high degree of steric hindrance within the monomeric SSADH structure, contributing to the structural destabilization of this variant [17]. Our studies show that the Cys93Phe substitution not only impairs the enzyme activity but also profoundly perturbs the protein expression of this variant, consistent with the predicted structural aberrations and destabilization caused by this substitution.

Asn255 is a highly conserved residue within the NAD^+^ binding domain, and the Asn255Asp substitution causes a rearrangement of hydrogen bonds that are required for the stabilization of a central β-sheet. Consistently, the enzyme activity and the protein level were very low, and the variant was insoluble in bacterial expression systems, pointing to a profound structural destabilization by the Asn255Asp substitution. In addition to a local effect within the NAD^+^ binding domain, this substitution is also likely to cause further structural rearrangements within the polypeptide chain, which may even result in destabilization or loss of the tetrameric SSADH structure. Notably, several previously described, severe pathogenic variants also map in this region, including Gly252Cys, Gly252Val, Asn255Ser, and Gly268Glu [15,17,24]. However, the Asn255Ser variant was shown to exhibit 17% of the WT SSADH activity in a transient overexpression system [15], but its protein expression level has not been studied. It is possible that the Ser substitution of Asn255 shows a milder structural perturbation than Asn255Asp, but its molecular consequences should be verified in a stable, low-level overexpression system, such as that used in our study and by Pop et al. [16].

In our SSADH-D patient, the Asn255Asp substitution was found in the same allele together with a His180Tyr amino acid exchange, resulting in an exchange of two amino acids within the SSADH polypeptide. The His180Tyr variant alone has a very mild effect on SSADH activity and shows a normal protein level (present study), with similar findings from previous studies [15,21]. Thus, this variant, found with a high frequency within the population [20], is, per se, not pathogenic. However, as shown previously, it may exacerbate the effect of further missense variants found within the same polypeptide chain, as shown previously for the Pro182Leu variant alone (44% residual activity) and in combination with His180Tyr (36% activity) [15]. 

In the case of our SSADH-D patient, the Asn255Asp substitution alone is already very severe, and His180Tyr does not further perturb the activity or expression of this variant. In eukaryotic expression systems, very low activity and protein levels are detected for the His180Tyr/Asn255Asp variant, similar to the single Asn255Asp substitution. In fact, even though the Asn255Asp recombinant SSADH protein was insoluble in bacteria, the double-substituted His180Tyr/Asn255Asp species was stable and could be structurally assessed in our study. The His180Tyr/Asn255Asp variant exhibits a highly decreased affinity for NAD^+^, which can be attributed to the Asn255Asp substitution. Therefore, the low activity of this variant as a recombinant protein is probably due to inefficient cofactor binding, whereas in eukaryotic cells, the protein expression is also disturbed.

As we observed a profound reduction of SSADH protein expression for the Cys93Phe and His180Tyr/Asn255Asp variants, and as our structural data show that these variants cause severe changes in the protein structure and possibly also in the tetramerization, it is likely that the low SSADH level in the patient cells is caused by accelerated degradation of the misfolded variant polypeptides. It is also possible, but less likely, that impaired protein translation might result in reduced SSADH levels, but there is so far no experimental evidence pointing in this direction. Nevertheless, this possibility should be assessed in future studies, as it may be important for the development of personalized therapies. 

We here show that the reason for the high, WT-like SSADH activity of the His180Tyr variant is due to the fact that it does not cause a major structural perturbation. His180 resides within a region that is involved in the tetramerization of SSADH monomers. The hydrogen bond formed by His180 through a bridging water molecule with Lys192’ residue in an adjacent monomer is retained in the His180Tyr variant, as the hydroxyl group of Tyr180 is at a hydrogen-bonding distance from Lys192’ of the neighboring subunit. However, further amino acid exchanges in the same polypeptide chain, e.g., Pro182Leu, may result in additional structural defects, such as impaired tetramerization. 

As a mitochondrial protein, SSADH is transported in an unfolded state into the mitochondria and only folds after reaching the matrix. The folding of mitochondrial proteins is supported by molecular chaperones that belong to the Hsp family [35]. Therefore, we attempted to increase the expression of Hsp70 proteins with two clinically relevant compounds, arimoclomol and celastrol [36,37,38]. Arimoclomol and related compounds increase the expression of Hsp70 chaperones by prolonging the activation of a transcription factor, Heat-shock Factor 1 (HSF1), that upregulates the expression of Hsp70 proteins, which in turn are able to aid in the folding of polypeptides with pathogenic variants [36,39,40,41,42,43]. Celastrol exhibits a similar mode of action by affecting HSF1 activation by increasing its DNA binding capacity and by inducing HSF1 hyperphosphorylation and active trimer stabilization, which results in increased Hsp70 expression [44,45]. In addition to the Hsp70 inducers, betaine, a chemical chaperone that generally supports protein folding, was used. 

The rationale behind the treatment with these compounds was that they would be able to increase Hsp70 expression and thus support the folding of the pathogenic SSADH variants, which should result in increased SSADH activity. Unfortunately, none of these compounds showed a significant effect on the expression or activity of SSADH in patient fibroblasts and in the knock-in expression system. In patient fibroblasts, a very small but non-significant increase in the activity was observed after 48 h of treatment, but a clear increase in the protein amount was not visible. In the light of our structural data, the pathogenic variants result in profound structural changes that may be so severe that even in the presence of enhanced Hsp70 chaperones, the folding cannot be rescued, and the variant polypeptides are degraded. 

Considering that the Asn255Asp variant shows an impaired cofactor binding, it may not be possible to reactivate this variant. The Cys93Phe variant could theoretically be more amenable to folding aids, as Cys93 is buried deep within the SSADH structure, and its exchange is likely to cause a structural effect instead of directly impairing NAD^+^ binding. However, no significant improvement in activity was observed upon treatment with the three compounds. It is also possible that the compounds used were not able to reach mitochondria, thus being unable to stabilize SSADH folding. Therefore, further compounds should be tested that may be capable of supporting the expression and folding of specific pathogenic SSADH variants, such as bimoclomol, a substance related to arimoclomol [43]. However, as these substances are toxic to the cells at higher concentrations (at or above 1 µM), causing substantial cell death, their therapeutic range in patients is limited [36,41,43,46,47]. It should also be taken into consideration that increased overexpression of Hsp proteins is associated with malignancies, and care should thus be taken when considering Hsp-enhancing therapies over a long time [48,49].

In this study, we have assessed personalized treatment strategies for an SSADH-D patient based on detailed molecular characterization of the consequences of the pathogenic SSADH variants. Unfortunately, the variants in the patient turned out to result in severe structural changes that may not be amenable to strategies targeting the folding of the pathogenic variants. Nevertheless, our data paves the way for assessing further treatments for this patient, which may be direct (such as gene therapy or enzyme replacement therapy) or indirect (e.g., reduction of toxic substances like GHB), as discussed in [2,13]. In the era of personalized precision medicine, it is important that the patients and their variants are considered and characterized individually so that the most efficient and safest therapy can be provided for all patients.

## 4. Materials and Methods

### 4.1. SSADH Deficiency Patient

A 1.5-year-old male German SSADH deficiency patient from a non-consanguineous marriage was diagnosed with SSADH-D, and the family was found to exhibit the following genetic variants in the *ALDH5A1* gene: c.269T>C, p.Val90Ala (maternal); c.278G>T, p.Cys93Phe; c.538T>C (paternal), p.His180Tyr, and c.763A>G, p.Asn255Asp (both maternal). The parents provided a signed informed consent for the study. 

### 4.2. Reagents

Succinic semialdehyde (SSA), succinic acid (SA), oxidized (NAD^+^) and reduced (NADH) nicotinamide adenine dinucleotide, isopropyl-β-d-thiogalactopyranoside (IPTG), phenylmethylsulfonyl fluoride (PMSF), poly-L-lysine, betaine and SigmaFast inhibitor cocktail were purchased from Merck (Darmstadt, Germany). Anti-SSADH (cat. sc-390754) and anti-His antibodies (cat. sc-8036) were from Santa Cruz Biotechnology (Heidelberg, Germany). ROTI^®^Mount FluorCare DAPI and 2-mercaptoethanol were from Roth (Karlsruhe, Germany). Hygromycin B was from Applichem GmbH (Darmstadt, Germany), and MACSfectin^TM^ transfection reagent was from Miltenyi Biotech (Bergisch Gladbach, Germany). Puromycin, zeocin, Alexa Fluor 488-conjugated secondary anti-rabbit antibody, and Mitotracker Orange were from Invitrogen/Thermo Fischer Scientific GmbH (Darmstadt, Germany). Further chemicals were of the highest purity available.

### 4.3. Site-Directed Mutagenesis of SSADH Expression Constructs

Human *ALDH5A1* open reading frame (ORF) and 36 bases of the 5’ untranslated region were cloned from pCDNA3 into pcDNA5/FRT vector (Invitrogen, Darmstadt, Germany). The constructs were used as templates for PCR-based, site-directed mutagenesis using the Quick-Change II Mutagenesis Kit (Agilent Technologies, Frankfurt am Main, Germany) to generate the SSADH missense variants Val90Ala, Cys93Phe; His180Tyr; Asn255Asp; and His180Tyr/Asn255Asp. The oligonucleotides used for the mutagenesis are shown in Table 1. All constructs were confirmed by DNA sequencing of the complete ORF. 

The ORF of human SSADH, without the mitochondrial targeting sequence (amino acids 1–47), was cloned with NdeI and XhoI recognition sequences into pET15b (Novagen, Life Science/Merck; Darmstadt, Germany), immediately adjacent to the N-terminal His-tag and the thrombin cleavage sequence. The oligonucleotides used for the mutagenesis are shown in Table 2. The constructs were verified by sequencing.

### 4.4. Eukaryotic Cell Culture

Human embryonic kidney (HEK-293T) and Flp-In^TM^-293 cells (Invitrogen/Thermo Fischer Scientific) were maintained in Dulbecco’s modified Eagle’s medium (DMEM) supplemented with 10% fetal calf serum (FCS) and 1% penicillin/streptomycin (all from Thermo Fisher Scientific). Cell cultures were maintained at 37 °C in a humidified incubator with 8% CO_2_. Patient-derived skin fibroblasts of the SSADH deficiency patient harboring the above-described variants were obtained from a punch biopsy. Immortalized healthy human skin fibroblasts were used as a control [50]. All fibroblasts were cultured in DMEM (high glucose) medium supplemented with 10% FCS, 1% penicillin/streptomycin, 1% non-essential amino acids, and 1% sodium pyruvate (all from Thermo Fischer Scientific), and grown at 37°C in a humidified incubator with 8% CO_2_.

### 4.5. Generation of an SSADH-Deficient ALDH5A1 Knockout Flp-In^TM^-293 Cell Line

*ALDH5A1* knockout cells (single-cell clones) were generated using the same strategy as described previously [22]. Briefly, gRNAs for the exon 3 of the human *ALDH5A1* gene (NM_001080.3) were cloned in the PX459 vector (Addgene: Cat.Nr.: 48139, Watertown, MA, USA). The gRNAs with the sequences 5′-CACCGGATGACTGCAGCCACGCCTA-3′ (fwd) and 5′-AAACTAGGCGTGGCTGCAGTCATCC-3′ (rev) were designed using the E-Crisp design tool [51]. The vector with the gRNA was used for the transfection of Flp-In^TM^-293 cells. The cells were first seeded onto 6-well plates in DMEM medium supplemented with 10% FCS and 1% penicillin/streptomycin and transfected with 1 μg SSADH-gRNA-PX459 and MACSfectin^TM^ reagent (Miltenyi Biotec) according to the manufacturer’s instructions. After 24 h, the cells were supplemented with 2 μg/mL puromycin and cultured for additional 48 h. The surviving cells were then seeded as single cells onto 96-well plates and expanded for further analysis. The knockout was confirmed by Western blot and PCR-based sequencing of the genomic DNA. 

### 4.6. Generation of Stable SSADH Knock-in Cell Lines

Flp-In^TM^-293 cell lines expressing WT SSADH or one of the SSADH variants (Cys93Phe, Asn255Asp, His180Tyr, or Asn255Asp/His180Tyr) were generated according to the Flp-In system manual (Thermo Fischer Scientific). The Flp Recombination Target (FRT) site present in the genome of the Flp-In^TM^-293 cells and in the expression vector pcDNA5/FRT allows for a stable genomic integration of the gene of interest by means of a Flp recombinase-mediated DNA recombination. The FRT site in Flp-In^TM^-293 cells is inserted downstream of a lacZ-Zeocin fusion gene. After the recombination, the cells lose the zeocin resistance and gain a hygromycin resistance, which can be used for the selection of single-cell clones. The SSADH-deficient Flp-In^TM^-293 *ALDH5A1* knockout cells were cotransfected with the pOG44 plasmid (expressing the Flp-recombinase) and the pcDNA5/FRT vectors with the SSADH variants (9:1 ratio). At 48 h post-transfection, the cells received 100 μg/mL hygromycin for additional 4 days. The hygromycin-resistant colonies were seeded onto 12-well plates, expanded, and analyzed further by western blot and PCR-based sequencing of the genomic DNA.

### 4.7. Transient Overexpression

Two hundred thousand HEK-293T and HEK-293T *ALDH5A1*-KO cells were seeded onto 6-well tissue culture plates in DMEM medium supplemented with 10% FCS and 1% Penicillin/Streptomycin. On the next day, the cells were transfected with 1 µg of pcDNA3 carrying either WT SSADH or Val90Ala, Cys93Phe, His180Tyr, Asn255Asp, or the double His180Tyr/Asn255Asp SSADH variant using MACSfectin^TM^ reagent (Miltenyi Biotec) according to the manufacturer’s protocol. After 48 h, the cells were solubilized in lysis buffer (50 mM Tris pH 7.4; 150 mM NaCl; 2 mM ethylenediamine-tetraacetic acid (EDTA); 1% NP-40), and the protein concentration was determined by Bradford assay (Biorad, Hercules, CA, USA) according to the manufacturer’s instructions.

### 4.8. Treatment of the Cells with Betaine and Hsp-Inducing Agents

SSADH-deficient *ALDH5A1* knockout Flp-In^TM^-293 cells stably expressing one of the SSADH variants and patient-derived fibroblasts were seeded onto 6-well culture plates and next day treated with 50 µM arimoclomol (Biosynth, Staad, Switzerland), 10 mM betaine (Sigma-Aldrich, Taufkirchen, Germany) or 250 nM celastrol (Selleckchem, Cologne, Germany). After 24 h or 48 h, the cell pellets were frozen at −20 °C. The concentrations and time points used in our study were chosen on the basis of literature findings and experimental data from our own laboratory showing positive results on other proteins [32,36,41,44,45,46,47].

### 4.9. Western Blotting

Prior to western blot, the cells were lysed in a lysis buffer (50 mM Tris pH 7.4; 150 mM NaCl; 2 mM EDTA; 1% NP-40) supplemented with a protease inhibitor cocktail (Sigma Aldrich). Protein lysates (1 µg) were separated on a 10% SDS-PAGE under reducing conditions, followed by transfer to a nitrocellulose membrane. After blocking the membrane with 5% milk powder (Roth, Karlsruhe, Germany) in TBST, the membrane was probed with one of the following primary antibodies: anti-SSADH (1:10,000, cat. ab 129017, Abcam, Cambridge, MA, USA) and anti-GAPDH (1:10,000, cat. ab-8245, Abcam). Thereafter, the membrane was incubated with horseradish peroxidase-labeled secondary antibodies (Dako, Gostrup, Denmark). The final detection of proteins was performed using an Enhanced Chemiluminescence kit (SuperSignal™ West Femto/Pico Chemiluminescent Substrate, Thermo Fisher Scientific). The signals were detected with an Odyssey^®^ XF Imaging System (LI-COR Biotechnology, Bad Homburg, Germany). The intensity of the SSADH signal was normalized to GAPDH.

### 4.10. SSADH Enzyme Activity

SSADH enzyme activity in the stable knock-in cell clones and their parental cells was analyzed by a fluorimetric assay as described previously [22], with the following modifications. Five hundred thousand cells were seeded onto a 6-well plate and solubilized in lysis buffer (50 mM Tris, pH 7.4; 150 mM NaCl; 2 mM EDTA; 1% NP-40) one day later. The protein lysates (1 µg) were pipetted in duplicates into a flat-bottom 96-well plate and incubated overnight at 37°C with 90 µL of an enzyme reaction mix containing 90 mmol/L Tris, pH 8.4, 0.2 mmol/L SSA, and 3 mmol/L NAD^+^. The SSADH-mediated reduction of NAD^+^ to NADH was measured as an increase in absorbance at 340 nm. To deduce the activity of other NAD^+^-reducing enzymes, the baseline values of samples incubated with a reaction mix without SSA were subtracted. Fluorescence was measured at an excitation wavelength of 355 nm and an emission wavelength of 470 nm with a TECAN Infinite 200 Microplate Reader (Tecan Group, Männedorf, Switzerland). 

SSADH activity in the patient-derived fibroblasts was analyzed similarly, with modifications of the lysis procedure. Fibroblasts were first scraped into H_2_O, supplemented with 0.2 mM PMSF (Fluka Chemicals, Buchs, Switzerland) and 0.1% 2-mercaptoethanol (Roth), and then sonicated (12 s at 90% amplitude) with Sonopuls Sonicator (Bendelin, Berlin, Germany). Ten µg of the protein lysate was used to measure SSADH activity.

### 4.11. Immunocytochemistry 

For SSADH staining, fifty thousand fibroblasts were seeded on poly-L-lysine coated glass coverslips in a 12-well plate and incubated on the following day for 30 min at 37 °C with fresh medium containing 100 nM MitoTracker Orange (Invitrogen) to stain mitochondria. The cells were washed and fixed with ice-cold methanol for 10 min at −20 °C, then blocked with 1% bovine serum albumin (BSA) in PBS for 30 min at room temperature, followed by a 1 h incubation with a rabbit anti-SSADH antibody (1:100, ab129017, Abcam). After washing, the cells were incubated with Alexa Fluor 488-conjugated secondary anti-rabbit antibodies (Invitrogen). The coverslips were subsequently mounted onto specimen slides with ROTI^®^Mount FluorCare DAPI (Carl Roth, Karlsruhe, Germany), and the samples were then imaged using an Aurox Clarity laser-free, spinning-disc confocal microscope (Aurox Ltd., Oxfordshire, UK) and the Visionary software (Version 4.2.10, Aurox Ltd., Oxfordshire, UK). Image processing was performed using the ImageJ software (Version 1.54h; NIH, Bethesda, MD, USA).

### 4.12. Bioinformatic Analyses 

Conservation analyses were performed with the Consurf Server [52] using the human SSADH amino acid sequence (isoform 2) and default values with a maximum of 150 homologs and an E-value cut-off of 0.0001 (E-value describing the number of expected hits). A conservation score from 1 (variable residue) to 9 (conserved residue) was attributed to each residue. BindProfX [25] was used to predict changes in the binding affinity upon the amino acid substitutions in the form of ΔΔG (change in free energy of binding) values. The algorithm combines the FoldX physics-based potential with the conservation scores from pairs of protein-protein interaction surface sequence profiles. By default, BindProfX will only look for highly similar interfaces (IS-score > 0.55) among known protein complex structures. The IS-score is given as a range from 0 to 1, with a higher value indicating a closer interface structure similarity, whereby an IS-score cut-off of 0.55 gives the best prediction. The human SSADH crystal structure (PDB 2W8N) was superimposed with a structure obtained by in silico mutagenesis of the selected residues by the software PyMOL (Version 2.2.3, Schrödinger, Limited Liability Company; LLC; New York, NY, USA).

### 4.13. Expression, Purification, and Enzymatic Assays of the Recombinant SSADH Species

Prokaryotic SSADH expression constructs in the pET15b vector (see Section 4.3) were transformed in *E. coli* BL21 cells. Bacteria were grown at 37°C in a selective Luria-Bertani broth with 0.1 mg/mL of ampicillin. Protein expression was allowed to proceed overnight at 30 °C after induction with 0.1 mM IPTG. The bacterial pellet, collected by centrifugation, was resuspended in a buffer (20 mM sodium phosphate, 20 mM imidazole, 500 mM sodium chloride, 10 mM β-mercaptoethanol, 0.5 mM PMSF, SIGMAFAST™ EDTA-free protease inhibitor cocktail, pH 7.40). Cell lysis was performed for 30 min at room temperature under constant agitation after adding lysozyme to a final concentration of 0.2 mg/mL. The suspension was then frozen in liquid nitrogen. After thawing, the suspension was treated with DNAse for 30 min under constant agitation, centrifuged, and the supernatant was loaded into a 5 mL HisTrap™ fast flow column (Cytiva, Global Life Sciences Solutions, Marlborough, MA, USA). A 50 min elution gradient was carried out for a single-step purification (flow 1 mL/min), with a second buffer containing 20 mM sodium phosphate, 500 mM imidazole, 500 mM sodium chloride, 10 mM β-mercaptoethanol, pH 7.40. The protein fractions were collected and dialyzed in 100 mM potassium phosphate, 10 mM β-mercaptoethanol, pH 8.00. The activity was measured spectroscopically at 340 nm as the NADH formed in the reaction (εM = 6220 M^−1^cm^−1^) following the addition of an enzymatic sample to a total of 0.35 mL of 10 µM SSA and 500 µM NAD^+^ in 100 mM potassium phosphate, 10 mM β-mercaptoethanol, pH 8. The recombinant SSADH (amino acids 48–535) was then concentrated with Spin-X UF concentrators (Corning Inc., New York, NY, USA) with cut-off 10 kDa, supplemented with 5% glycerol, frozen in liquid nitrogen, and finally stored at −80 °C. SDS-PAGE was performed using 12% polyacrylamide gel to assess the purity of the recombinant SSADH. To assess the oligomerization state, size exclusion chromatography was carried out in 100 mM potassium phosphate and 150 mM NaCl, pH 8. Different concentrations (from 0.1 to 5 mg/mL) of recombinant WT and His180Tyr SSADH were loaded into a Superdex 200 (10/300) column (GE Healthcare, Boston, MA, USA) coupled to an ÄKTA Pure system (GE Healthcare), and the 280 nm signal was monitored. 

### 4.14. Spectroscopic Analyses and Determination of the Equilibrium Dissociation Constant for NAD^+^

All spectroscopic measurements were carried out at 25 °C in 100 mM potassium phosphate buffer, pH 8, unless otherwise stated. Absorbance spectra were recorded with a Jasco V-550 spectrophotometer (Jasco Europe S.R.L., Milano, Italy), whereas intrinsic fluorescence emission analyses were carried out in a Jasco FP-750 spectrofluorimeter (Jasco Europe S.R.L) at 0.1 mg/mL SSADH, with 5 nm excitation and emission bandwidths upon excitation at 280 nm. Circular dichroism (CD) spectra were acquired in a Jasco J-710 spectropolarimeter (Jasco Europe S.R.L) at a scan speed of 50 nm/min with 2 nm bandwidth, using 0.1 mg/mL and 1 mg/mL protein concentrations for the far UV (190–250 nm) and near UV/visible (250–550 nm) range, respectively. Thermal denaturation was performed by monitoring the CD signal at 222 nm of 0.1 mg/mL SSADH species on a 25–90 °C linear temperature gradient with a temperature slope of 1.5 °C/min.

The equilibrium dissociation constant, K_D_, for NAD^+^ was calculated by plotting the change in the intrinsic fluorescence emission, following excitation of the enzyme at 280 nm, as a function of NAD^+^ concentration and fitting it to the following equation:

$$Y=Y_{max}\times \frac{\left(\left[E\right]+\left[NAD^{+}\right]+K_{D}\right)-\sqrt{{\left(\left[E\right]+\left[NAD^{+}\right]+K_{D}\right)}^{2}-\left(4\times \left[E\right]\times \left[NAD^{+}\right]\right)}}{2\times \left[E\right]}$$

where Y refers to the change in intrinsic fluorescence at each NAD^+^ concentration, Y_max_ to the maximal change in the intrinsic fluorescence at the saturating NAD^+^ concentration, and [E] refers to the monomeric SSADH concentration.

### 4.15. Statistical Analysis

Statistical analysis was performed using the GraphPad Prism software (v. 5.02, La Jolla, CA, USA). The data are presented as mean values ± SD unless otherwise stated. Differences between the two groups were tested using Student’s *t*-test. A comparison of multiple groups was performed by one-way ANOVA followed by Tukey’s post-hoc test.

## Figures and Tables

**Table 1:** Oligonucleotide sequences used for PCR-based site-directed mutagenesis of eukaryotic expression vectors. All sequences are shown in 5′–3′ direction. F: forward, R: reverse.

Substitution	Sequence
Val90Ala	F: CGCTCTGGGCATGGCAGCCGACTGCGGGG R: CCCCGCAGTCGGCTGCCATGCCCAGAGCG
Cys93Phe	F: GGGCATGGTAGCCGACTTCGGGGTGCG R: CGCACCCCGAAGTCGGCTACCATGCCC
His180Tyr	F: GTGTTTACGGAGACATTATCTACACCCCGGCAAAG R: CTTTGCCGGGGTGTAGATAATGTCTCCGTAAACAC
Asn255Asp	F: GATTCCTTCAGGTGTATACGATGTTATTCCCTGTTCTCG R: CGAGAACAGGGAATAACATCGTATACACCTGAAGGAATC

**Table 2:** Oligonucleotide sequences used for PCR-based site-directed mutagenesis of prokaryotic expression vectors. All sequences are shown in 5′–3′ direction. F: forward, R: reverse.

Substitution	Sequence
Val90Ala	F: GCTGGGCATGGCTGCGGATTGCGGTG R: CACCGCAATCCGCAGCCATGCCCAGC
Cys93Phe	F: GCATGGTTGCGGATTTCGGTGTTCGTGAAGC R: GCTTCACGAACACCGAAATCCGCAACCATGC
His180Tyr	F: GTTTATGGTGACATCATTTACACCCCGGCGAAGG R: CCTTCGCCGGGGTGTAAATGATGTCACCATAAAC
Asn255Asp	F: GCGGCGTTTACGACGTGATTCCGTGCAG R: CTGCACGGAATCACGTCGTAAACGCCGC

## Data Availability

The original research data are available from the authors upon a reasonable request.

## Supplementary Materials

The following supporting information can be downloaded at https://www.mdpi.com/article/10.3390/ijms25105237/s1. Reference [53] is cited in Supplementary Materials.
