# Supplementary material for: Functional Characterization of a Spectrum of Genetic Variants in a Family with Succinic Semialdehyde Dehydrogenase Deficiency
Source: Int J Mol Sci. 2024 May 11;25(10):5237. doi: 10.3390/ijms25105237 (PMC11121183; doi:10.3390/ijms25105237)
Supplement: Supplementary file 1 [file ijms-25-05237-s001.zip › ijms-2973494-supplementary.pdf]

Supplementary Table S1

Table S1. Secondary structure percentage of WT and H180Y evaluated by BeStSel [1], and T_M of the same species measured at 222 nm.

#	α-Helix (%)	β-Sheet (%)	Others (%)	T_M 222 nm (°C)
WT	28.9	23.0	48.1	52.98 ± 0.05
WT + 200 μM NAD^+	24.6	27.9	47.5	57.5 ± 0.7
H180Y	30.6	25.9	43.5	49.2 ± 0.2
H180Y + 200 μM NAD^+	30.0	13.1	56.9	53.7 ± 0.5

Reference

1. Micsonai, A.; Moussong, E.; Wien, F.; Boros, E.; Vadaszi, H.; Murvai, N.; Lee, Y.H.; Molnar, T.; Refregiers, M.; Goto, Y.; Tantos, A.; Kardos, J. BeStSel: webserver for secondary structure and fold prediction for protein CD spectroscopy. *Nucleic Acids Res* **2022**, *50*, W90-W98, 10.1093/nar/gkac345.

Suppl. Figure S1

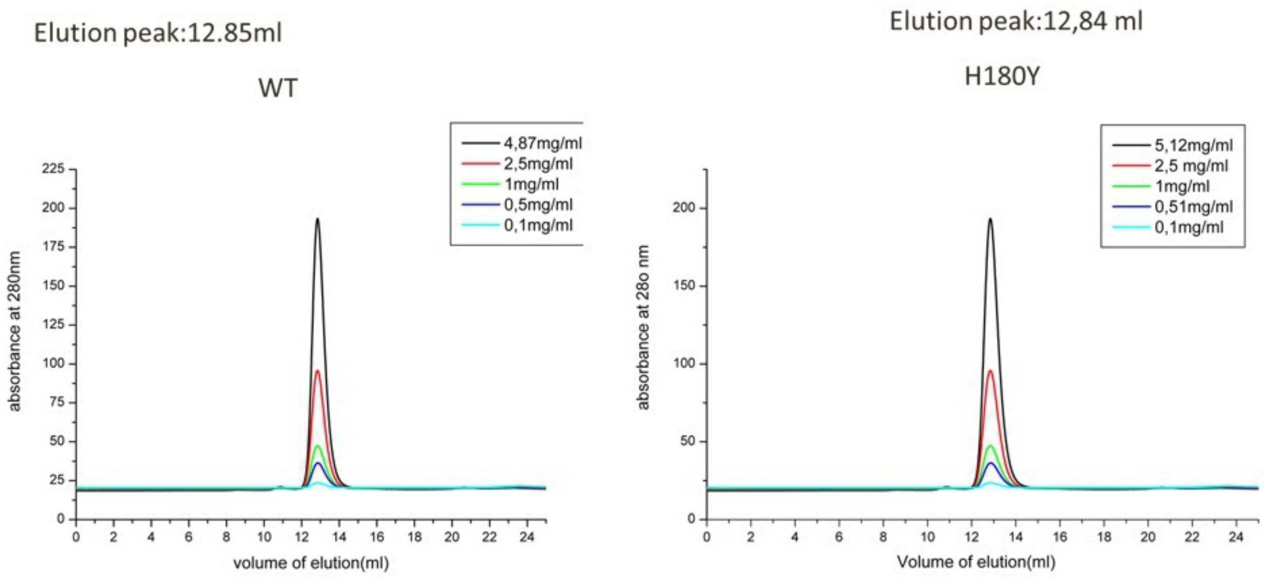

Supplementary Figure S1: Molecular size of WT (left) and His180Tyr (right) SSADH. Size exclusion chromatography was carried out in 100 mM potassium phosphate and 150 mM, NaCl pH 8, at the indicated SSADH concentrations reported as mg/mL. One mg corresponds to 18 units for the WT and 15 units for H180Y variant (one unit of activity is defined as the amount of enzyme required to convert 1 mmol/min of NAD^+ to NADH).
